# Positive Peer Group Affiliation, Puberty, and Social and Emotional Development: A Strength‐Based Approach

**DOI:** 10.1002/jad.70205

**Published:** 2026-06-15

**Authors:** Rona Carter, Joonyoung Park

**Affiliations:** ^1^ Department of Psychology University of Michigan Ann Arbor MI USA

**Keywords:** emotional development, positive peer relationships, pubertal status, social

## Abstract

**Introduction:**

During the pubertal transition, peer relationships become increasingly salient for adolescents' social and emotional development. However, research on peer contexts during this period has largely emphasized deviant peer groups, leaving the potential role of positive peer affiliations less understood. The present study examined whether positive peer group affiliation mediated associations between pubertal development and social and emotional development.

**Methods:**

Data were drawn from 1364 youth in the United States (48% female; 76% White; *M* age = 9.32 years, SD = 0.48 at Wave 3) participating in Grades 4 through 6 of Phase III of the NICHD Study of Early Child Care and Youth Development. Latent growth curve models were used to examine associations among pubertal timing and tempo, positive peer group affiliation, and social and emotional development.

**Results:**

Exploratory sex‐stratified estimates suggested that earlier pubertal timing was associated with lower initial levels of social and emotional development among boys, but not girls. Across groups, positive peer group affiliation was associated with higher initial levels of social and emotional functioning. Mediation models indicated that pubertal timing was associated with lower positive peer group affiliation for boys, whereas pubertal tempo was associated with declines in peer affiliation over time for girls. However, there was limited evidence that pubertal development or peer affiliation predicted change in social and emotional development over time.

**Conclusions:**

Findings suggest that positive peer group affiliation is linked to concurrent social and emotional functioning but does not appear to mediate longitudinal associations between puberty and adjustment.

1

For many children, pubertal development is not only a biological transition but also a socially consequential one. Youth who mature earlier than their peers often encounter shifts in expectations, social roles, and peer dynamics at a time when many same‐age peers have not yet undergone similar changes. As a result, early‐developing youth may experience a sense of mismatch between themselves and their peer group, which has been linked to a range of behavioral and emotional difficulties (Mendle et al. 2006; Negriff and Susman 2011).

A substantial body of research has sought to explain these associations by focusing on peer processes, particularly affiliation with deviant peers. Studies consistently show that early pubertal timing is associated with increased likelihood of affiliating with peers who engage in problem behaviors, which in turn is linked to maladjustment (Lynne et al. 2007; Marceau and Jackson 2017; Mendle and Ferrero, 2012; Mendle et al. 2012; Mrug et al. 2014; Stepanyan et al. 2020; Teunissen et al. 2011; Wang et al. 2020; Zhu et al. 2016). For example, Lynne et al. (2007) demonstrated that early‐developing youth who affiliated with deviant peers exhibited elevated levels of aggressive and delinquent behavior across middle school.

Despite this emphasis, far less is known about the role of positive peer group affiliation, that is, affiliation with peers who display prosocial characteristics such as kindness, cooperation, and reliability. This omission is notable, given that peer affiliation reflects not only exposure to risk but also access to protective social resources. A growing body of work suggests that affiliating with prosocial peers is associated with lower levels of problem behavior and greater social competence (Busching and Krahe, 2020; Denio et al. 2020; Donlan et al. 2015; Gallardo et al. 2016; Haddow et al. 2021; Lansford et al. 2003; Williams and Anthony 2015). Thus, understanding how positive peer group affiliation is linked to pubertal development may provide a more balanced account of the social processes surrounding puberty. Importantly, peer affiliation is not random. Rather, it reflects processes of selection and social organization. Youth tend to form relationships with peers who are similar to themselves—a principle known as homophily (Brown, 2004; McPherson et al. 2001). In the context of pubertal development, youth who mature earlier or more rapidly may seek out peers who appear more similar in physical development, even if those peers differ in chronological age (Haynie 2003; Lynne et al. 2007; Negriff et al. 2011; Stattin et al. 2011). These selection processes may shape the peer contexts in which early‐developing youth are embedded. However, similarity may also emerge around behavioral and social characteristics, such as prosociality. From this perspective, peer affiliation may reflect both maturational alignment and shared social orientations.

## 2 Guiding Theoretical Framework

Theoretical support for the current study is drawn from the contextual amplification hypothesis (see Ge and Natsuaki 2009). According to this hypothesis, contextual conditions (e.g., interpersonal challenges, disapproval among peers) can attenuate or intensify the effects of early pubertal development on psychosocial outcomes through the implicit reward and punishment structures provided by social contexts (e.g., family conflict, neighborhood, interpersonal experiences). Adaptation is expected to be particularly difficult for children in adverse peer contexts, such as those who affiliate with deviant peers (Ge et al. 2002; Compian et al. 2009; Hamlat et al. 2015; Nadeem and Graham 2005). However, the risk of adverse outcomes is decreased for early‐developing children in supportive environments. Of interest to the present study is the context and people with which children are directly and frequently in contact—schools and peers.

Theoretical accounts of peer processes also guide our investigation of the associations between positive peer group affiliation, puberty, and social and emotional development. Homophily is the principle that social connections foster and are fostered by shared similarities (McPherson et al. 2001). Accordingly, children who develop early or faster relative to their same‐age and sex peers may select friends of comparable physical development rather than chronological age (Haynie 2003; Kretsch e al., 2016; Lynne et al. 2007; Negriff et al. 2011; Stattin et al. 2011). Because older adolescents engage in deviant behaviors that are relatively normative for them (Sampson and Laub 2003) but non‐normative for early and faster developers, the selection and influence of older peers may make early developing children vulnerable to developing social and emotional problems over time. However, using this same line of reasoning, children may select friends based on shared values (e.g., prosociality) and thus reduce the risk for social and emotional problems.

## 3 Peer Affiliation and Puberty‐Linked Outcomes

Research on peer processes during adolescence has largely focused on problem behaviors. Youth who affiliate with peers who engage in deviant behavior are more likely to exhibit externalizing outcomes themselves (e.g., Brendgen et al. 2000; Mason et al. 2019; Patterson et al. 2000; Vitaro et al. 2000; Wang et al. 2020). In contrast, youth who affiliate with prosocial peers tend to show lower levels of problem behavior and higher levels of adaptive functioning (e.g., Fabes et al. 2012; Stotsky et al. 2020). For example, children high in prosocial behavior are more likely to associate with similarly prosocial peers and less likely to interact with aggressive or antisocial peers (Fabes et al. 2012). Despite these patterns, relatively little research has examined whether positive peer group affiliation is linked to pubertal development and its social‐emotional correlates. It remains unclear whether early pubertal development is associated with reduced access to prosocial peer networks, whether such affiliations are linked to social and emotional functioning, and whether peer processes help explain associations between puberty and adjustment.

Another important consideration is the role of informants. Much of the existing literature relies on youth or parent reports of behavior, yet findings may differ depending on who is reporting. For example, Carter et al. (2009) found that associations between pubertal timing and behavioral outcomes varied across teacher, parent, and youth reports. Because youth function across multiple contexts, incorporating multiple informants—particularly teachers who observe youth in structured peer environments—may provide a more comprehensive understanding of how pubertal development is linked to social functioning.

## 4 The Current Study

**Figure 1:**
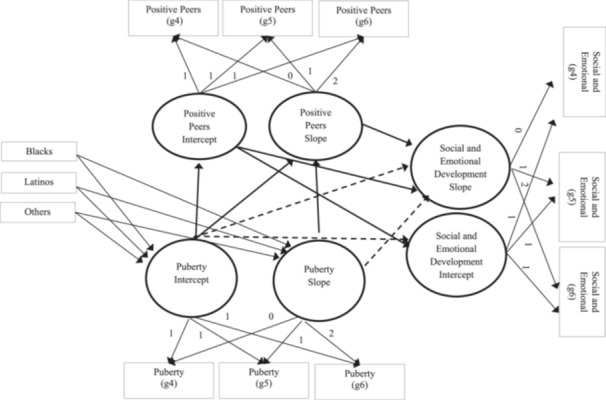
Latent growth curve multiple mediation model for pubertal status, positive peer affiliation, and social and emotional development with race\ethnicity and SES as covariates for pubertal status.

Drawing on the contextual amplification hypothesis and theories of peer processes, the present study examined associations among pubertal development, positive peer group affiliation, and social and emotional development during early adolescence (Grades 4–6). Using latent growth curve modeling, we focused on both initial levels and changes over time in these constructs, see Figure 1. Specifically, we examined whether pubertal development (timing and tempo) was associated with (a) positive peer group affiliation and (b) social and emotional development, and whether positive peer group affiliation was associated with social and emotional functioning. We also tested whether positive peer group affiliation statistically accounted for associations between pubertal development and social and emotional development. Given the limited research on positive peer processes in the context of puberty, particularly with respect to longitudinal change, we approached these analyses as an empirical test of potential pathways rather than specifying strong directional hypotheses. We also examined sex‐specific patterns within a multigroup framework. Given mixed findings in prior literature and because sex differences were not a primary focus of the study, these analyses were exploratory. Accordingly, we did not frame the study as a formal test of sex moderation, and sex‐specific estimates are interpreted descriptively.

## 5 Method

### 5.1 Data Source and Sample

Data were drawn from families who participated in the National Institute of Child Health and Human Development study of Early Childcare and Youth Development (NICHD SECCYD), one of the few studies examining pubertal development while simultaneously examining positive peer group affiliation and social and emotional development. NICHD SECCYD is a prospective longitudinal study conducted from 1991 through 2006 at ten research sites across the United States. These data have been used effectively in many child and adolescent development studies. Of the 8,986 mothers who gave birth during selected 24‐h sampling periods, 5,416 (60%) agreed to be telephoned in 2 weeks and met the eligibility requirements (i.e., mother was over 18, spoke English, was healthy, had a single baby who was not released for adoption and lived within an hour of a research site in a neighborhood not deemed too dangerous by police to visit). One thousand five hundred twenty‐five families were selected for the call and agreed to an interview. Of these, 1364 completed a home interview when the infant was 1 month old and became study participants. The recruited sample consisted of 52% boys, 24% of participants of color, 11% of mothers not completing high school, and 14% of single‐parent families. The present study used data from the children and families from the recruited sample followed from 4th through 6th grade (Phase III). In fourth grade, fifty‐eight percent of the mothers were married, 18% were separated/divorced, 3% lived with a partner, and 21% were missing/not reported. Families earned $81,035 on average a year. Sixty‐one percent of the mothers were White, 9% American Indian, 8% Black, 4% Latina, 3% Asian, and 15% were missing.

### 5.2 Procedures

Details about all data collection procedures, the instrument's psychometric properties, and composites' descriptions are documented in the study's Manuals of Operation and Instrument Documentation (http://secc.rti.org). Institutional Review Board approval was obtained for this study.

### 5.3 Measures

**Positive peer group affiliation.** Teachers' perceptions of children's peer relationships (Relationships with Peers: Teacher Version ‐ Part D‐9‐items) were assessed in grades 4–6. Sample items include “These kids are often in trouble” and “These kids are kind to other kids.” Teachers rated children's peer group affiliation on a 5‐point Likert scale from 1 *(definitely no)* to 5 *(definitely yes*). A child's positive peer group score is computed as the sum of items 1–6 and 8–10 after reflecting items 2–5. This score was imputed by proportional weighting. Scores calculated in this manner have a possible range from 9 to 45 and an actual range from 11 to 45, with higher scores indicating more positive peer group affiliation. Cronbach's *α* across grades was above 0.91, indicating acceptable reliability.

**Pubertal development.** Mothers completed the 5‐item Pubertal Development Scale (PDS; Petersen et al. 1988) in grades 4–6 to assess the extent to which children experienced pubertal growth in several domains during the past 12 months. On a scale ranging from 1 (*have not begun*) to 4 (*development completed*), mothers rated children of both sexes on their body hair development, growth spurt, and skin changes. Mothers rated the development of breasts and the occurrence of menarche for daughters and the development of facial hair and voice changes for sons. The PDS yields a composite score by averaging the five items within each sex (range = 1–4), with higher scores indicating more advanced pubertal development. Cronbach's *α* for boys and girls across grades was above 0.60, indicating acceptable reliability. Past research has established satisfactory predictive validity for the PDS with self‐rated Tanner Stage, Kappa = 0.50 (Bond et al. 2006). The average PDS total scores for this sample of fourth graders were 1.44 (SD = 0.34) for girls and 1.26 (SD = 0.23) for boys, suggesting that their pubertal development had just begun.

**Social and emotional development.** Teachers' judgments of children's social skillfulness with peers in grades 4–6 were assessed using seven items from the Teacher Checklist of Peer Relations (Coie and Dodge 1988). On a scale ranging from 1 (very poor) to 5 (very good), teachers rated the following: Understands others' feelings, Is socially aware of what is happening in a situation, Accurately interprets what a peer is trying to do, Refrains from over impulsive responding, Generates many solutions to interpersonal problems, Generates reasonable quality solutions to interpersonal issues, and Is aware of the effects of their behavior on others. A score for social and emotional development was obtained by summing responses across the seven items, with higher scores indicating high social and emotional development levels. Cronbach's *α* across grades was above 0.90, indicating acceptable reliability.

**Control variables.** Given that pubertal development varies significantly by race/ethnicity (Herman‐Giddens et al. 1997), ethnic‐racial group membership in 4th grade was included as a time‐invariant covariate for pubertal development. Race was reflected in the models by 3 dummy variables coded for Blacks (1 = Black and 0 = all else), Latinos (1 = Latino and 0 = all else), and Others (1 = Other and 0 = all else). The omitted (reference) group in all analyses is Whites. Socioeconomic status was also included as a covariate, given that studies indicate its impact on pubertal timing.

### 5.4 Data Analyses Plan

Latent growth curve (LGC) multiple mediation analysis was used to examine the associations between pubertal development and social and emotional growth, highlighting the mediating role of positive peer group affiliation. This analytic approach can provide information about the relative advantage of applying the initial status of the putative mediator, its change, or both factors to explain the relationship between the independent and dependent variables. The advantage of using such multiple mediation models is that by including both the intercept and the slope of the putative mediator variable as predictors, mutual control for the effects of each of the two variables on the dependent variable is ensured (von Soest and Hagtvet 2011).

Longitudinal measures of pubertal development were modeled with a latent factor representing the initial level of development (i.e., intercept) and a latent factor representing a linear change in development (i.e., slope). The intercept factor represents pubertal timing. Children with a higher intercept have higher pubertal status at the earliest observation age (i.e., they are early developers relative to their peers). The slope factor for pubertal development represents pubertal tempo; children with a higher slope are predicted to increase in pubertal status more quickly over time. Longitudinal changes in social and emotional development and positive peer group affiliation were also modeled using linear growth models. Time scores were coded as years since baseline (T1 = 0; T2 = 1; T3 = 2), such that intercepts reflect levels at Grade 4.

Mplus version 7 (Muthén and Muthén 2010) examined the hypothesized relationships between pubertal development, social and emotional development, and positive peer group affiliation. The following equations can mathematically express the LGC mediation model:

$$\propto_{i\omega }=\mu_{\alpha \omega }+\gamma_{\alpha \omega }x_{i}+\zeta_{\alpha \omega i}$$

$$\beta_{i\omega }=\mu_{\beta \omega }+\gamma_{\beta \omega }x_{i}+\zeta_{\beta \omega i}$$

$$\beta_{\mathrm{iy}}=\mu_{\beta y}+\delta_{\alpha \omega }\alpha_{i\omega }+\delta_{\beta \omega }\beta_{i\omega }+\gamma_{\beta y\prime }x_{i}+\zeta_{\beta \mathrm{yi}}$$

Analyses proceeded in a stepwise fashion to facilitate model interpretation. First, unconditional LGC models were estimated separately for pubertal development, positive peer group affiliation, and social and emotional development to determine the shape of the developmental trajectory of these variables. In fitting the unconditional models, significant variance in intercept would indicate substantial individual differences in these variables at baseline (i.e., 4th grade). Considerable variation in latent growth factors (slope) would show individual differences in change over time for these variables. In each case of these linear models, the fit indexes were acceptable (CFI > 0.95; RMSEA < 0.08, and SRMR close to 0.08), and variances of the intercept and slope were significant (*p* < 0.05).

Next, a multivariate LGC model was estimated to examine associations between pubertal development and social and emotional development. Consistent with temporal ordering, pubertal timing (intercept) was specified as a predictor of both baseline levels (intercepts) and change (slopes) in social and emotional development. Pubertal tempo (slope), reflecting change over time, was specified to predict only change (slope) in social and emotional development. Paths from pubertal tempo to baseline (intercept) outcomes were not estimated due to temporal ordering constraints.

To examine mediation, positive peer group affiliation was introduced as a putative mediator using a parallel process LGC multiple mediation framework (von Soest and Hagtvet 2011). Both the intercept and slope of positive peer group affiliation were included to assess whether mediation operates through baseline levels, change over time, or both. Consistent with temporal ordering, pubertal timing (intercept) was specified to predict both intercept and slope factors of positive peer group affiliation and social and emotional development. Pubertal tempo (slope) was specified to predict only slope factors of the mediator and outcome variables. The intercept and slope of positive peer group affiliation were specified to predict the intercept and slope of social and emotional development, respectively. In addition, the slope of social and emotional development was adjusted for its intercept to account for baseline differences (Seltzer et al. 2003). Indirect effects were estimated to evaluate mediation pathways, and bias‐corrected bootstrap confidence intervals were used to determine statistical significance.

Ethnicity‐race was included as a covariate predicting pubertal timing and tempo. Ethnicity‐race was represented using three dummy‐coded variables: Black (1 = Black, 0 = all others), Latino (1 = Latino, 0 = all others), and Other (1 = Other, 0 = all others), with White youth serving as the reference group. Socioeconomic status (SES) was also included as a covariate using three dummy‐coded variables, given its established association with pubertal development and adjustment. SES was specified as a predictor of intercept factors for pubertal development, positive peer group affiliation, and social and emotional development.

Because prior findings regarding sex differences in puberty‐linked peer and adjustment processes are mixed, sex‐specific estimates were examined exploratorily within the multigroup framework. Boys and girls were modeled simultaneously; however, the primary purpose of this approach was to estimate sex‐specific pathways rather than to conduct a formal test of sex moderation. Thus, we did not conduct nested model comparisons or equality‐constraint tests across sex. Consistent with this exploratory approach, we avoid interpreting significant associations in one group and nonsignificant associations in the other as evidence that parameters differ significantly across groups.

**Model Estimation and Evaluation.** A robust weighted least squares estimator was used in all analyses (Muthén 1984). Missing data were handled using full information maximum likelihood (FIML), which provides unbiased parameter estimates under missing at random assumptions. Model fit was evaluated using multiple indices, including the comparative fit index (CFI), root mean square error of approximation (RMSEA), and standardized root mean square residual (SRMR). Model fit was considered acceptable when CFI ≥ 0.95, RMSEA ≤ 0.08, and SRMR ≤ 0.08 (Kline 2011). Chi‐square statistics were also reported, with the acknowledgment that they are sensitive to sample size. Indirect effects were estimated using bias‐corrected bootstrap confidence intervals based on 5,000 resamples (Hayes 2009; Shrout and Bolger 2002).

**Missing data.** Missing data across measures and time points ranged from 25% to 35% and were primarily due to attrition and incomplete assessments. Missing‐data bias was evaluated by creating dummy variables for each variable indicating missingness and examining their associations with study variables. No systematic patterns of missingness were detected, supporting the assumption that data were missing at random. Given the extent and pattern of missingness, FIML estimation was used to retain all available data and minimize bias.

## 6 Results

### 6.1 Descriptive Statistics

Table 1 presents means, standard deviations, and correlations among all study variables. Social and emotional development and positive peer group affiliation demonstrated moderate stability across the study period (Grades 4–6).

**Table 1:** Means, variances, and correlations for all study variables.

	1	2	3	4	5	6	7	8	9
1. SED g4	0.81								
2. SED g5	0.67**	0.80							
3. SED g6	0.59**	0.65**	0.79						
4. PDS g4	−0.02	−0.01	−0.01	0.09					
5. PDS g5	−0.01	−0.01	0.01	0.82**	0.18				
6. PDS g6	−0.02	−0.01	0.03	0.65**	0.82**	0.30			
7. PPAg4	0.51**	0.07*	0.03	−0.04	−0.01	0.05*	42.73		
8. PPA g5	0.13**	0.08*	−0.03	−0.02	0.02	0.03	0.36**	44.44	
9. PPA g6	0.05	0.11*	0.12*	0.05	0.07*	0.42**	0.42**	0.42**	47.46
Means	3.56	3.63	3.62	1.35	1.56	1.85	37.42	37.19	36.82

Abbreviations: PDS, pubertal development scale; PPA, positive peer group affiliation; SED, social and emotional development; variances are on the diagonal.

* *p* <0.01;

** *p* <0.05.

### 6.2 Univariate Latent Growth Curve Models

Unconditional (univariate) latent growth curve (LGC) models were estimated separately for social and emotional development, positive peer group affiliation (prosocial behavior), and pubertal development. A multigroup framework was used, with boys and girls modeled simultaneously.

Social and Emotional Development. The unconditional linear growth model demonstrated acceptable fit, *χ* ^2^(2) = 3.46, *p* = 0.17, CFI = 0.99, RMSEA = 0.04 (90% CI [0.01, 0.10]), SRMR = 0.02. Among boys, the mean intercept was significant (*M* = 3.38, SE = 0.04, *p* < 0.001), with significant variability (*V* = 0.56, SE = 0.07, *p* < 0.001), indicating individual differences in baseline levels. The mean slope was not significant (*M* = 0.02, SE = 0.02, *p* = 0.276), and slope variability was also not significant (*V* = 0.06, SE = 0.04, *p* = 0.117). Among girls, the mean intercept was also significant (*M* = 3.78, SE = 0.04, *p* < 0.001), with significant variability (*V* = 0.38, SE = 0.06, *p* < 0.001). The mean slope was statistically significant but small (*M* = 0.04, SE = 0.02, *p* = 0.036), and slope variability was not significant (*V* = 0.03, SE = 0.03, *p* = 0.292). These findings indicate meaningful individual differences in baseline levels of social and emotional development for both boys and girls, but limited variability in rates of change over time.

Positive Peer Group Affiliation (Prosocial Behavior). The unconditional growth model demonstrated excellent fit, *χ* ^2^(2) = 0.51, *p* = 0.774, CFI = 0.99, RMSEA = 0.01 (90% CI [.01, 0.06]), SRMR = 0.01. Among boys, the mean intercept was significant (*M* = 35.90, SE = 0.31, *p* < 0.001), with significant variability (*V* = 14.89, SE = 5.01, *p* = 0.003). The mean slope was not significant (*M* = −0.33, SE = 0.20, *p* = 0.092), and slope variability was not significant (*V* = −1.47, SE = 2.83, *p* = 0.603). Among girls, the mean intercept was significant (*M* = 39.23, SE = 0.24, *p* < 0.001), with marginal variability (*V* = 6.57, SE = 3.51, *p* = 0.061). The mean slope was not significant (*M* = −0.13, SE = 0.16, *p* = 0.416), and slope variability was not significant (*V* = −0.30, SE = 2.14, *p* = 0.889). Overall, these findings indicate substantial individual differences in baseline levels of positive peer group affiliation, but little evidence of systematic change or variability in change over time.

Pubertal Development. The unconditional growth model demonstrated relatively good fit, *χ* ^2^(2) = 39.19, *p* < 0.001, CFI = 0.98, RMSEA = 0.17 (90% CI [.12, 0.21]), SRMR = 0.02. Although RMSEA was elevated, other indices suggested acceptable fit. Among boys, both the intercept (*M* = 1.26, SE = 0.01, *p* < 0.001; *V* = 0.05, SE = 0.07, *p* < 0.001) and slope (*M* = 0.17, SE = 0.01, *p* < 0.001; *V* = 0.01, SE = 0.01, *p* = 0.001) were significant, indicating variability in pubertal timing and tempo. Among girls, both intercept (*M* = 1.44, SE = 0.01, *p* < 0.001; *V* = 0.11, SE = 0.01, *p* < 0.001) and slope (*M* = 0.34, SE = 0.01, *p* < 0.001; *V* = 0.05, SE = 0.01, *p* = 0.001) were also significant. These findings indicate meaningful individual differences in both pubertal timing and tempo across sexes Table 2.

**Table 2:** Standardized parameter estimates from the latent growth curve multiple mediation model of pubertal development, peer exclusion, and social competence.

	Boys (*n* = 705)		Girls (*n* = 659)	
Model parameters	Estimate	95% CI	Estimate	95% CI
Growth factors				
Puberty intercept	[0.00]		[0.00]	
Puberty slope	0.17*	[0.15, 0.19]	0.34*	[0.32, 0.36]
Positive peers intercept	0.78*	[0.70, 0.85]	0.81*	[0.74, 0.87]
Positive peers slope	−0.08	[−0.20, 0.04]	−0.03	[−0.14, 0.08]
Social and emotional intercept	0.75*	[0.68, 0.82]	0.79*	[0.72, 0.85]
Social and emotional slope	0.02	[−0.03, 0.07]	0.04	[0.01, 0.07]
Structural paths				
Puberty timing → Intercept positive peers	−0.29*	[−0.45, −0.13]	−0.07	[−0.21, 0.07]
Puberty timing → Slope positive peers	0.39*	[0.15, 0.63]	0.02	[−0.18, 0.22]
Puberty tempo → Slope positive peers	0.05	[−0.10, 0.20]	−0.21*	[−0.39, −0.03]
Intercept positive peers → Intercept social and emotional	0.99*	[.94, 1.04]	0.91*	[0.85, 0.97]
Puberty timing → Intercept social and emotional	0.06	[−0.09, 0.21]	−0.02	[−0.14, 0.10]
Puberty timing → Slope social and emotional	0.01	[−0.10, 0.12]	−0.02	[−0.11, 0.07]
Puberty tempo → Slope social and emotional	0.03	[−0.09, 0.15]	0.04	[−0.07, 0.15]
Intercept positive peers → Slope social and emotional	0.02	[−0.08, 0.12]	0.03	[−0.06, 0.12]
Slope positive peers → Slope social and emotional	0.05	[−0.09, 0.19]	0.07	[−0.08,0.22]
Social and emotional intercept→ Slope	−0.38*	[−0.65, −0.11]	−0.12	[−0.30, 0.06]
Indirect effects (Bootstrapped)				
Intercept for social and emotional	−0.05	[−0.12, 0.02]	−0.01	[−0.43, −0.03]
Slope for social and emotional	0.01	[−0.08, 0.10]	−0.03	[−0.05, 0.05]

*Note:* 95% CI = bias‐corrected bootstrap confidence intervals. Sex‐specific estimates were examined exploratorily. Formal equality‐constraint tests comparing boys and girls were not conducted; therefore, differences in significance across groups should not be interpreted as evidence of statistically significant sex differences.

* *p* < 0.05 (based on bias‐corrected bootstrap confidence intervals).

### 6.3 Multivariate Growth Model Without Mediation

A multigroup multivariate LGC model was estimated to examine associations among pubertal development, positive peer group affiliation, and social and emotional development. Boys and girls were modeled simultaneously. The model demonstrated adequate but not optimal fit, *χ* ^2^(142) = 462.27, *p* < 0.001, CFI = 0.89, TLI = 0.86, RMSEA = 0.07 (90% CI [0.06, 0.08]), SRMR = 0.12.

Among boys, earlier pubertal timing was associated with lower initial levels of social and emotional development (*b* = −0.21, SE = 0.10, *p* = 0.034, β = −0.11). Pubertal timing was also associated with a change in social and emotional development (*b* = 0.14, SE = 0.07, *p* = 0.033), although the standardized estimate was not significant, suggesting a small and unstable effect.

Pubertal tempo was not associated with change in social and emotional development (*p* = 0.314).

Initial levels of positive peer group affiliation were strongly associated with initial levels of social and emotional development (*b* = 2.61, SE = 0.27, *p* < 0.001). No associations were observed with change over time.

Among girls, pubertal timing was not associated with initial levels of social and emotional development (*b* = −0.09, SE = 0.07, *p* = 0.179) or change over time (*ps* > 0.50). Pubertal tempo was also not associated with change (*p* = 0.726). Although the association between positive peer group affiliation and social and emotional development was significant in unstandardized form (*b* = 1.46, SE = 0.21, *p* < 0.001), standardized estimates suggested this effect was less stable and should be interpreted cautiously.

Summary of Multivariate Model. Overall, exploratory sex‐specific estimates suggested that pubertal timing, but not tempo, was modestly associated with initial levels of social and emotional development among boys. A comparable association was not observed among girls. Because formal equality constraints were not tested, this pattern should not be interpreted as evidence of a statistically significant sex difference. Positive peer group affiliation was strongly associated with baseline social and emotional functioning, particularly among boys. Across both groups, there was little evidence that pubertal development or peer affiliation predicted change over time, consistent with limited slope variability observed in the univariate models.

### 6.4 Multivariate Growth Model With Mediation

A multigroup LGC model was estimated to examine whether positive peer group affiliation mediated associations between pubertal development and social and emotional development. The model demonstrated acceptable fit, *χ* ^2^(136) = 383.07, *p* < 0.001, CFI = 0.92, TLI = 0.89, RMSEA = 0.06 (90% CI [0.06, 0.07]), SRMR = 0.12.

Among boys, earlier pubertal timing was associated with lower initial levels of positive peer group affiliation (*b* = −3.15, SE = 0.95, *p* = 0.001). Positive peer group affiliation was strongly associated with initial social and emotional development (*b* = 0.17, SE = 0.02, *p* < 0.001). No predictors were associated with change over time. Among girls, pubertal timing was not associated with peer affiliation or social and emotional development. However, pubertal tempo was associated with decreases in positive peer group affiliation over time (*b* = −0.77, SE = 0.34, *p* = 0.022). Positive peer affiliation was again associated with baseline social and emotional functioning. Tests of indirect effects revealed no evidence of mediation for either boys or girls (all *ps* > 0.65). This pattern was consistent across standardized and unstandardized estimates.

Summary of Mediation Model. Taken together, results do not support positive peer group affiliation as a mediator linking pubertal development to social and emotional development. Instead, findings suggest that peer affiliation and social functioning are closely aligned at baseline, but pubertal development does not appear to influence social and emotional trajectories through peer processes over time.

### 6.5 Sensitivity Analyses

Sensitivity analyses using alternative indicators and raters of pubertal development (mother and nurse‐reported Tanner stages; Tanner, 1971) yielded largely consistent patterns of results Although some path estimates varied, the overall conclusions remained unchanged: associations were primarily observed at baseline, with limited evidence of longitudinal mediation.

## 7 Discussion

Although affiliation with deviant peers is frequently emphasized as a key mechanism linking early pubertal development to adjustment, far less is known about the role of positive peer group affiliation. The present study extended existing work by examining whether affiliations with prosocial peers were associated with links between pubertal development and social and emotional development during early adolescence. Drawing on longitudinal data from the NICHD Study of Early Child Care and Youth Development and using a multi‐reporter design, we examined these processes across Grades 4 through 6.

Several findings are noteworthy. First, the results offered only limited support for a direct association between pubertal development and social and emotional development. In the multivariate model without mediation, earlier pubertal timing was associated with lower initial levels of social and emotional development for boys, but not for girls. Pubertal tempo was not associated with social and emotional development for either group. These findings suggest that pubertal timing may be modestly related to early social‐emotional functioning under some conditions, but that these associations were not robust across models or across sex.

Second, positive peer group affiliation was consistently and strongly associated with social and emotional development at baseline. Across models, youth with higher levels of positive peer group affiliation tended to show higher initial levels of social and emotional functioning. This pattern was especially clear for boys and was also evident, though somewhat less stable, for girls. These findings align with prior research on the importance of positive peer relationships for children's adjustment (Denio et al. 2020; Donlan et al. 2015; Gallardo et al. 2016; Haddow et al. 2021; Lansford et al. 2003; Williams and Anthony 2015), and they underscore that affiliating with prosocial peers is closely tied to social‐emotional competence during early adolescence.

Third, despite these strong baseline associations, the mediation model did not support positive peer group affiliation as an indirect pathway linking pubertal development to social and emotional development. For boys, earlier pubertal timing was associated with lower initial levels of positive peer group affiliation, and positive peer group affiliation was associated with higher initial levels of social and emotional development. However, indirect effects were not significant. For girls, pubertal timing was not associated with initial levels of peer affiliation, although a faster pubertal tempo was associated with decreases in peer affiliation over time. Again, these patterns did not translate into significant indirect effects. Taken together, the results are consistent with past research on peer affiliation and pubertal development (Mendle and Ferrero, 2012; Stepanyan et al. 2020; Teunissen et al. 2011; Yu et al. 2022). The findings also suggest that positive peer group affiliation is an important correlate of early social‐emotional functioning, but it did not function as a longitudinal explanatory mechanism in the present models.

This distinction matters conceptually. The findings suggest that peer relationships may reflect, rather than transmit, the developmental correlates of pubertal maturation during this age period. That is, puberty and peer context may both be linked to social‐emotional functioning, but the data do not support the conclusion that positive peer group affiliation carries the effect of pubertal development forward into later change in social and emotional development. This is an important boundary condition for theories that position peer processes as a central pathway through which puberty shapes adjustment.

The absence of significant growth effects is also informative. Across the unconditional models, there was limited variability in slopes for both positive peer group affiliation and social and emotional development. Youth differed meaningfully in their baseline levels of peer affiliation and social‐emotional functioning, but they showed relatively similar trajectories of change across Grades 4 through 6. This restricted variability likely constrained the ability to detect longitudinal associations involving slope factors in the multivariate and mediation models. More substantively, the pattern suggests that puberty in this developmental window may operate more as a marker of early social positioning than as a driver of diverging developmental trajectories. Early differences in peer context and functioning may matter more than subsequent differences in rate of change, at least across late childhood to early adolescence.

These findings still highlight the importance of positive peer contexts. Even though mediation was not supported, the robust association between positive peer group affiliation and baseline social and emotional development suggests that prosocial peer networks may serve as an important social resource during early adolescence. Youth who affiliate with supportive, prosocial peers may have greater access to encouragement, modeling of adaptive behaviors, and opportunities for positive social engagement (Laursen and Veenstra, 2021; Wagner, 2019). In this sense, positive peer affiliation appears less like a conveyor belt carrying pubertal effects forward over time and more like a social anchor tied closely to concurrent adjustment.

At the same time, the present findings suggest caution in assuming that all theoretically plausible peer mechanisms operate longitudinally in the same way. Much of the puberty literature has emphasized deviant peer affiliation as a developmental risk pathway. The present study suggests that positive peer affiliation deserves attention as well, but perhaps not only or primarily as a mediator. Instead, it may be more useful to think of positive peer group affiliation as an indicator of a broader social ecology (e.g., empathy & optimism; Oberle et al. 2010) that co‐occurs with stronger adjustment in early adolescence.

Because sex differences were exploratory and not a primary aim of the study, we did not conduct formal nested model comparisons or equality‐constraint tests across sex. Accordingly, the sex‐specific patterns observed here should be interpreted descriptively rather than as evidence of sex moderation. Although the estimates suggested that pubertal timing was more consistently associated with baseline social‐emotional functioning and positive peer affiliation among boys, whereas pubertal tempo was associated with declining peer affiliation among girls, these patterns should not be taken to indicate that the corresponding parameters differed significantly across groups. Future research designed specifically to test sex moderation should formally compare constrained and unconstrained multigroup models.

### 7.1 Limitations and Future Directions

Several limitations should be considered when interpreting these findings. First, although the study used longitudinal data, the strongest associations emerged at the level of intercepts, reflecting baseline levels rather than change over time. Accordingly, the most interpretable findings are largely cross‐sectional in nature, and causal inferences should be made cautiously. Future studies should prioritize designs and measures that allow stronger tests of longitudinal processes and mediation over time. Second, there was limited variability in slope factors for positive peer group affiliation and social and emotional development, and some slope‐related estimates in the multivariate models were unstable. These features limited the ability to draw strong conclusions about developmental change and suggest that the present data may be better suited to understanding baseline differences than longitudinal divergence.

Third, although socioeconomic indicators were included as covariates, future research should continue to investigate how structural conditions shape pubertal experiences, peer contexts, and adjustment. Puberty does not unfold in a social vacuum, and socioeconomic conditions may influence both when youth mature and how those changes are interpreted by others (Carter et al. 2024). Fourth, the sample was predominantly White, which limits generalizability. The social meaning of pubertal development likely varies across racialized, ethnic, and cultural contexts, and pubertal changes may be interpreted differently depending on broader stereotypes, social expectations, and community norms (Natsuaki, 2013) Future work should examine these questions in more diverse samples and explicitly consider how race, ethnicity, and context shape the peer ecology of puberty.

Fifth, the measure of positive peer group affiliation, while useful, does not capture the full complexity of peer experiences. Future research would benefit from examining additional dimensions of peer functioning, including friendship quality, peer acceptance, peer support, and peer influence processes. It may be that more specific peer constructs are better suited to explaining how puberty becomes socially consequential. Finally, future studies should consider whether specific visible indicators of pubertal development, such as height changes, breast development, voice changes, or body composition, are especially salient in peer contexts. Some pubertal changes may be more likely than others to alter peer relationships or social evaluations.

## 8 Conclusion

For some youth, puberty is not only a biological transition but also a socially meaningful experience. The present findings suggest that positive peer group affiliation is closely tied to social and emotional development during early adolescence, but it did not mediate associations between pubertal development and social‐emotional change over time. Rather than functioning as a longitudinal pathway, positive peer affiliation appeared to be most strongly linked to concurrent differences in adjustment. By centering positive peer contexts, this study broadens the conversation beyond risk‐based models of puberty and peer influence. Even without evidence of mediation, the findings highlight the value of understanding how supportive peer ecologies co‐occur with healthier functioning during the pubertal transition. Efforts to promote prosocial peer networks may still hold promise for supporting youth during this developmental period, particularly for those whose pubertal experiences are socially challenging.

## Author Contributions

**Rona Carter:** conceptualization, investigation, methodology, writing – review and editing, writing – original draft, formal analysis, project administration. **Joonyoung Park:** writing – review and editing, conceptualization.

## Conflicts of Interest

The authors declare no conflicts of interest.

## Data Availability

The data that support the findings of this study are openly available in the Inter‐university Consortium for Political & Social Research at https://www.icpsr.umich.edu/web/ICPSR/series/00233.
